# Three-Dimensional Displacement Patterns in Maxillary Molar Distalization: A Comparative Finite Element Study

**DOI:** 10.3390/dj14030187

**Published:** 2026-03-23

**Authors:** Roland Kmeid, Joseph Bouserhal, Allahyar Geramy, Maria Daccache, Moschos Papadopoulos

**Affiliations:** 1 Department of Orthodontics, Faculty of Dental Medicine, Saint Joseph University of Beirut, Rue de Damas, Beirut 1107 2180, Lebanon; 2 Department of Orthodontics, Boston University Henry M. Goldman School of Dental Medicine, 635 Albany Street, Boston, MA 02118, USA; 3 Department of Orthodontics, School of Dentistry, Tehran University of Medical Sciences, North Kargar Street, Tehran 1417614411, Tehran, Iran; 4 Department of Orthodontics, Faculty of Dentistry, School of Health Sciences, Aristotle University of Thessaloniki, 54124 Thessaloniki, Central Macedonia, Greece

**Keywords:** orthodontic anchorage procedures, tooth movement, finite element analysis, orthodontic appliances

## Abstract

**Objectives:** This study aimed to analyze the three-dimensional displacement of maxillary first molars using a finite element model with two headgear configurations, namely cervical and horizontal pull headgears, as well as pendulum, infrazygomatic miniscrews, Bollard miniplates, Advanced Molar Distalization Appliance (AMDA), and Beneslider. The goal was to clarify how variations in anchorage design and force direction influence molar movement across the sagittal, vertical, and transverse planes. **Methods:** A three-dimensional finite element model of the maxillary dentition and supporting structures was constructed using reference anatomical data and standardized material properties. Each appliance was virtually simulated under its clinically recommended force magnitude and direction to ensure realistic biomechanical conditions. The orientation of each force vector relative to the molar’s center of resistance (CR) was analyzed, and resulting tooth displacements were quantified along the sagittal (Z), vertical (Y), and transverse (X) axes using 49-node reference paths connecting key anatomical landmarks. **Results:** Appliances applying forces through or above the molar CR, such as the AMDA, infrazygomatic miniscrews, and Bollard miniplates, produced nearly bodily distalization with minimal tipping (<0.6° (range 0.3–0.6°)) and slight intrusion (−0.12 to −0.18 mm). Conversely, systems delivering forces below the CR, such as the cervical headgear and pendulum, resulted in greater crown tipping and extrusion. The Beneslider exhibited an intermediate displacement pattern with moderate vertical control. **Conclusions:** Force vector height and direction relative to the molar CR critically determine 3D displacement behavior. Skeletal anchorage and adjustable systems, particularly the AMDA, demonstrated the most controlled distalization pattern with minimal tipping, whereas conventional tooth-borne designs induced more tipping and extrusion.

## 1. Introduction

Class II malocclusion, one of the most prevalent orthodontic problems, affects up to 37% of European children and one-third of orthodontic patients in the United States [1,2,3]. Management depends on patient age and severity, with treatment options ranging from growth modification to camouflage and orthognathic surgery [4]. For non-extraction correction, maxillary molar distalization is frequently employed, as it creates space, corrects molar relationships, and reduces overjet without extractions [5,6,7].

Traditional distalization methods, such as extraoral headgear, can be effective, but their success is highly compliance-dependent, and many patients reject them for esthetic reasons [8]. To reduce reliance on cooperation, intraoral distalizers (e.g., the pendulum and distal jet) were introduced; however, they may be associated with molar tipping and anchorage loss [9,10]. More recently, skeletal anchorage devices, including miniscrews and miniplates, have addressed many of these limitations by providing substantially improved anchorage control compared with conventional tooth-borne mechanics [11,12,13].

Finite element analysis (FEA) enables three-dimensional evaluation of orthodontic biomechanics under controlled conditions, allowing for the isolation of mechanical factors and precise measurement of displacement vectors in the sagittal, vertical, and transverse planes [14,15]. Several FEM studies have reported that skeletal anchorage systems produce more bodily molar movement with less tipping compared to conventional systems [16,17,18].

Skeletal anchorage devices—including infrazygomatic miniscrews, miniplates, and prefabricated systems such as the AMDA^®^ and the Beneslider—have been developed to enhance anchorage control and facilitate more controlled en masse distalization with fewer unwanted side effects [11,19,20,21,22,23,24]. Comparative clinical studies generally suggest skeletal anchorage can achieve more bodily movement and greater efficiency than conventional tooth-borne approaches, although protocol heterogeneity persists [25,26]. FEA studies complement these findings by isolating appliance-specific biomechanical responses under standardized conditions [16,25,26].

Despite numerous clinical and finite element studies examining individual distalization appliances, there is still no standardized comparative analysis that evaluates both conventional and skeletal anchorage systems under identical geometric and loading conditions. Most previous investigations have focused on a single appliance or used differing model parameters, which makes it difficult to distinguish the specific effects of anchorage design and force vector orientation on three-dimensional tooth movement. Although the general direction of molar displacement can be qualitatively anticipated from the line of action and the center of resistance, a quantitative assessment of simultaneous sagittal, vertical, and transverse displacements remains scarce.

To address this gap, the present study compared six maxillary distalization appliances within a unified finite element framework, allowing a direct and standardized evaluation of their mechanical behavior. This approach is particularly valuable because understanding displacement not only in the sagittal but also in the vertical and transverse planes is essential for maintaining occlusal balance, periodontal health, and long-term treatment stability [27].

We hypothesized that appliances using skeletal anchorage and higher lines of force application would produce more controlled, bodily distalization and superior vertical management compared with conventional tooth-borne systems.

## 2. Materials and Methods

### 2.1. Study Design

**Figure 1:**
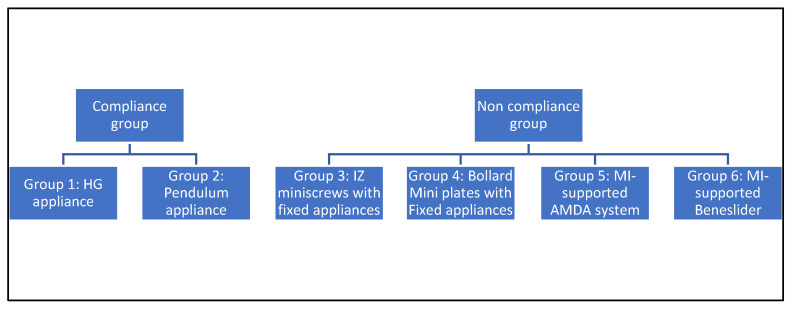
Diagram of the different appliance groups.

This three-dimensional finite element study compared six maxillary distalization appliances: cervical headgear, horizontal pull headgear, pendulum, infrazygomatic miniscrews, Bollard miniplates, AMDA^®^, and Beneslider (Figure 1).

### 2.2. Model Construction

The dentition was designed according to Ash’s dental anatomy [28] and then moved to form a dental arch shape. Maxillary dentition from the central incisor to the second molar was modeled in SolidWorks (version 2023; Dassault Systèmes, Vélizy-Villacoublay, France) and transferred to Ansys Workbench 2021 R1 (ANSYS, Version 18, Mechanical APDL, ANSYS Inc., Canonsburg, PA, USA) for the analysis. A uniform periodontal ligament (PDL) layer of 0.25 mm was incorporated around each root, along with surrounding cortical and trabecular bone. Although PDL thickness varies by root surface location and patient-specific anatomy, a uniform thickness was assumed to maintain standardized model geometry across appliances. Material properties for teeth, cortical and spongy bone, and the periodontal ligaments were all derived from average values reported in the literature. The contact elements were defined as bonded between the PDLs and root surfaces and between the spongy and cortical bones. All brackets were defined as bonded to the tooth crown surfaces. The contacts between the tube and wire were defined as frictional (*static friction coefficient 0.20*). The second molar was also included in the analysis with frictional contact with the first molar (*static friction coefficient 0.20*). The different components’ Young’s modulus and Poisson’s ratio values are listed in Table 1 [17,29]. Accordingly, the geometry represents an idealized maxillary dentoalveolar anatomy based on standard references rather than a patient-specific CBCT-derived segmentation, which allows for standardized inter-appliance comparison.

### 2.3. Appliances

**Figure 2:**
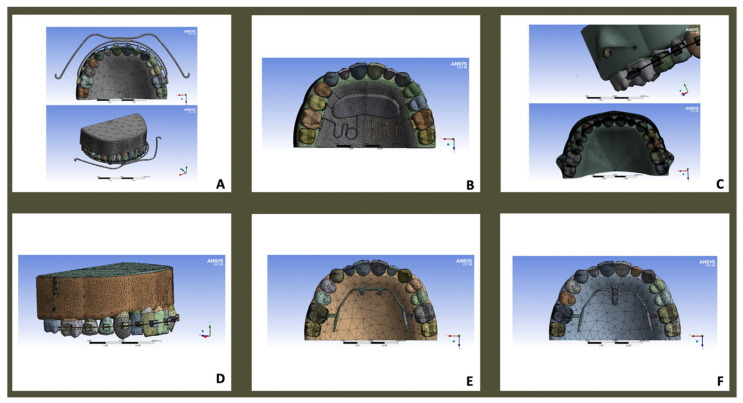
Finite element modeling of six maxillary molar distalization appliances: (**A**) headgear; (**B**) pendulum; (**C**) infrazygomatic miniscrews with fixed appliances; (**D**) Bollard miniplates with fixed appliances; (**E**) AMDA^®^; (**F**) Beneslider (Beneplate with miniscrews).

Each appliance was modeled virtually and loaded according to clinically reported force magnitudes, either on the palatal or buccal side of the first molar (Figure 2A–F, Table 2).

- Headgear (Figure 2A).
- Pendulum (Figure 2B).
- Infrazygomatic miniscrews with fixed appliances (Figure 2C).
- Bollard miniplates with fixed appliances (Figure 2D).
- AMDA^®^ (Figure 2E).
- Beneslider (Beneplate with miniscrews, Figure 2F).

The detailed mechanical characteristics, anchorage type, force application, and sequential versus en masse mechanics of each distalization approach are summarized in Table 3.

### 2.4. Force Application and Analysis

Forces were applied as summarized in Table 2. To preserve clinical realism, the applied force magnitudes were not standardized across all appliances but rather selected according to the values reported in the original clinical or experimental protocols specific to each device. The purpose of this comparative finite element analysis was not to equate mechanical loads artificially but to investigate how each distalization system behaves under its recommended clinical activation conditions. This approach allows the assessment of the intrinsic biomechanical characteristics of each design—reflecting the direction, point of application, and anchorage type unique to its clinical use—rather than the effect of a uniform external force. This analysis evaluates the immediate initial displacement following a single activation step and does not simulate sequential activation stages, cumulative distalization, or anchorage loss over time. Consequently, the reported displacement patterns are interpreted primarily in relation to variations in force vector geometry and anchorage configuration; however, the magnitude of displacement may still be influenced by the applied force level and should not be treated as a standalone metric of biomechanical efficiency across appliances. A mesh convergence analysis was also performed: the mesh was refined until further refinement caused negligible changes in the results.

### 2.5. Force Vector Geometry and Center of Resistance

Each distalization appliance delivers its force along a specific line of action relative to the center of resistance (CR) of the maxillary first molar, estimated to be located about 8–10 mm apical and 2 mm distal to the furcation. The orientation of this line of action largely determines whether the resulting movement is bodily, tipping, intrusive, or extrusive.

- Cervical Headgear: The line of force passes below the CR, directed distally and slightly downward. This low line of pull produces a clockwise moment, leading to distal crown tipping and a tendency for molar extrusion.
- Horizontal (High-Pull) Headgear: The force line acts closer to or slightly above the CR, which reduces the tipping moment and introduces a mild intrusive component compared with the cervical configuration.
- Pendulum Appliance: The palatal spring applies a posterior and slightly upward force from the palatal side; the resultant vector passes below and medial to the CR, producing distal crown movement, palatal root displacement, and a mild constrictive transverse effect.
- Infrazygomatic Miniscrews: The buccal coil springs generate force above and slightly outward from the CR, promoting near-bodily distalization with minimal tipping and a slight intrusive effect.
- Bollard Miniplates: The point of force application lies higher and more posterior than with miniscrews, directing the traction line through or slightly above the CR, which favors bodily movement and excellent vertical control.
- AMDA (palatal miniscrews): The palatal line of force is nearly horizontal but positioned above the CR, enabling controlled distalization with mild root intrusion.
- Beneslider: The bilateral palatal screws produce a horizontal force line at approximately the level of the CR, promoting balanced sagittal translation while minimizing vertical side effects.

Although both the AMDA and Beneslider rely on palatal miniscrew anchorage, they differ significantly in their ability to control the height and orientation of the applied force vector. The AMDA allows clinicians to choose among three distinct activation levels, corresponding to different spring attachment heights on the palatal screws. This adjustability permits fine-tuning of the line of action so that it can be directed through, above, or below the estimated CR of the molar. Such flexibility is particularly valuable because the CR varies among patients depending on root morphology, length, and bone support.

In contrast, the Beneslider’s design provides a fixed horizontal line of force at an almost constant height relative to the molar CR. While this geometry allows for efficient distalization, it offers less control over the vertical and rotational components of movement compared with the AMDA. Consequently, the AMDA’s adjustable vector geometry represents a biomechanical refinement that enables more individualized and bodily molar displacement.

The maxilla was constrained at the bispinal plane. Displacement was measured along sagittal (Z), vertical (Y), and transverse (X) planes. To test the numeric findings, two paths of nodes, each containing 49 nodes, were defined on the right first molar. The first path was between the mesio-buccal root apex and the mesio-buccal cusp tip to trace the mesio-distal displacements of the molar (apico-occlusal path). The second path was defined between the distal fossae of the first molar and the mesial fossae of the same tooth to assess the medio-lateral (expansion and constriction) displacements and also the supero-inferior (extrusion and intrusion) ones. This boundary condition was chosen to stabilize the model and prevent rigid-body motion; it simulates craniofacial skeletal stability and is commonly adopted in orthodontic finite element models evaluating molar distalization mechanics.

## 3. Results

The finite element analysis revealed distinct three-dimensional initial displacement patterns for the six appliances tested, with differences evident in the sagittal, vertical, and transverse planes. These findings underline the varying biomechanical characteristics of conventional and skeletal anchorage-based distalization strategies.

A path of nodes was defined using 49 nodes along these lines, facilitating a detailed analysis of displacement for the different distalization approaches. The finite element analysis (FEA) conducted along the apico-occlusal path, from the root apex (node 1) to the mesiobuccal cusp tip (node 49), allowed for the quantification of the sagittal displacements for the distalization appliances. Positive displacement values indicate distalization, whereas negative values correspond to mesialization.

**Figure 3:**
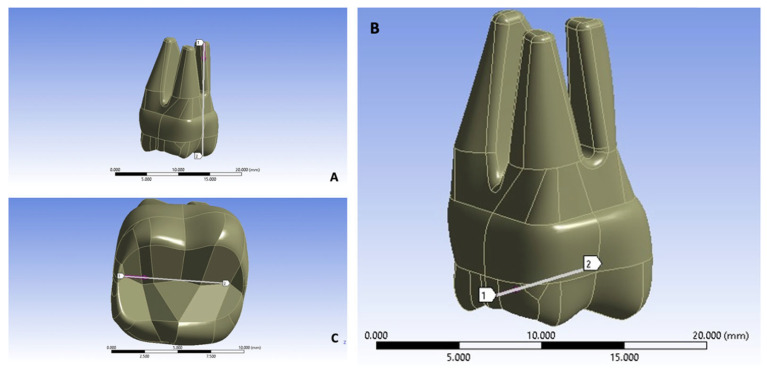
Measurement paths used to assess molar displacement: (**A**) mesio-distal (ΔZ) displacement; (**B**,**C**) vertical and transverse displacements.

The mesio-distal (∆Z) displacement of the upper molars was assessed along the apico-occlusal path, which connects the mesiobuccal apical point to the mesiobuccal cusp tip of the molar (Figure 3A), whilst the vertical and transverse displacements were assessed along the mesio-distal occlusal path, which connects the mesial and distal fossae on the occlusal surface of the molar (Figure 3B,C). Because the model geometry and loading conditions were symmetric, analysis of the right molar was considered representative of bilateral displacement behavior.

### 3.1. Sagittal Displacement

**Figure 4:**
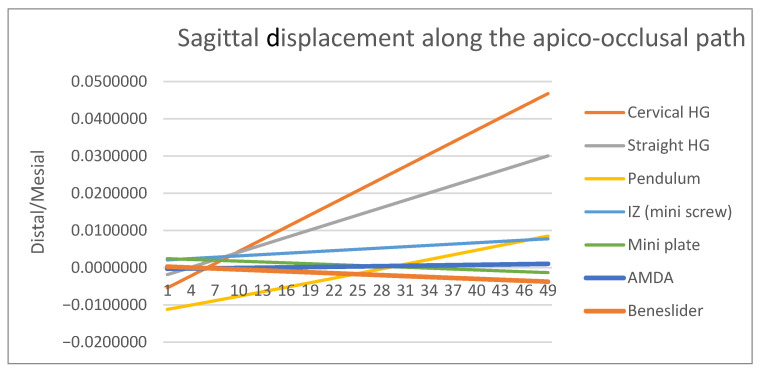
Sagittal displacement of the maxillary first molar along the apico-occlusal path [mm] across the six appliances tested (positive values: distalization; negative values: mesialization). Skeletal anchorage and AMDA approximated bodily movement, whereas the headgear and pendulum showed greater crown tipping.

All appliances were effective in producing distalization of the maxillary first molars; however, the degree of bodily control varied substantially (Figure 4, Table 4). The AMDA and Beneslider demonstrated favorable displacement patterns, with crown and root distalization occurring in a relatively parallel manner, thus approximating bodily movement. The cervical headgear produced the most pronounced distal crown tipping, indicating poor root control and highlighting its limitations in maintaining bodily displacement. The horizontal pull headgear provided somewhat better root control than the cervical configuration, but tipping remained evident. By contrast, skeletal anchorage devices—the miniscrews and the miniplates—offered the most favorable outcomes, with nearly parallel crown and root displacement and minimal tipping. The pendulum appliance, though effective in distalizing the crown, was limited by a lack of corresponding root displacement, producing a pronounced tipping effect. Overall, while all appliances achieved distalization, bodily movement was best approximated by the AMDA, infrazygomatic miniscrews, and miniplates, whereas the cervical headgear and pendulum demonstrated the highest tipping tendencies.

### 3.2. Vertical Displacement

**Figure 5:**
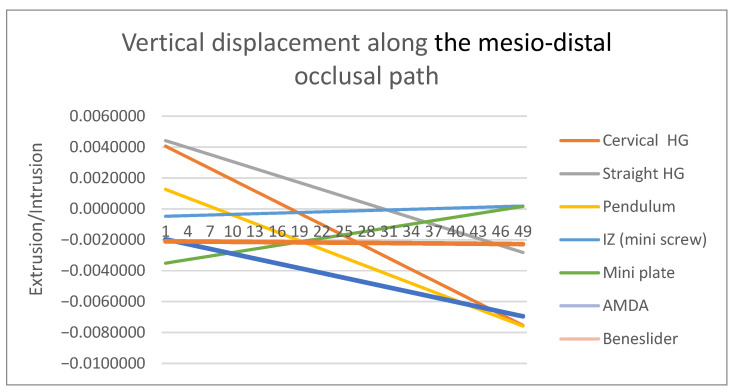
Vertical displacement patterns of the maxillary first molars along the mesio-distal occlusal path [mm] across the six appliances tested (positive values: intrusion; negative values: extrusion). Cervical headgear produced extrusion, while the pendulum and Beneslider revealed marked intrusion. The AMDA showed mild vertical displacement, and skeletal anchorage devices provided the most stable vertical control.

Vertical changes further distinguished the appliances (Figure 5, Table 5). The cervical headgear produced extrusion of the molar crown, an outcome that may be clinically unfavorable in hyperdivergent patients. The pendulum and Beneslider showed marked intrusion tendencies, consistent with their line of force application. The AMDA demonstrated mild vertical changes, primarily root intrusion with relatively stable crown positioning. Both skeletal anchorage systems exhibited excellent vertical control, as minimal displacement was detected. This stability underscores their value in patients where vertical dimension management is critical.

### 3.3. Transverse Displacement

**Figure 6:**
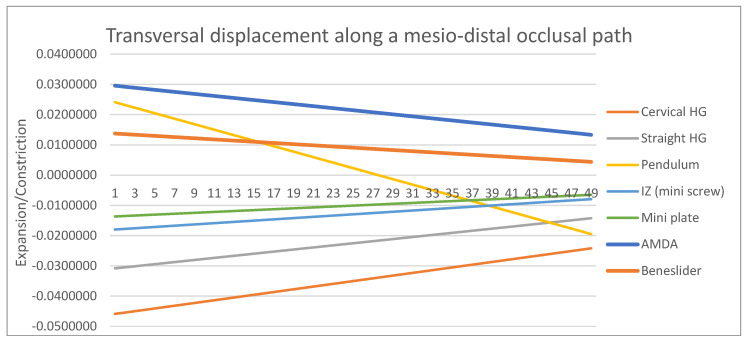
Transverse displacement patterns of the maxillary first molars along the mesio-distal occlusal path [mm] across the six appliances tested (positive values: constriction; negative values: expansion). Headgear appliances demonstrated buccal crown displacement, while the pendulum showed palatal root displacement. The AMDA and Beneslider maintained balanced control, and skeletal anchorage devices exhibited the most symmetrical and stable patterns.

Although transverse changes were less prominent than sagittal or vertical displacement, meaningful differences were observed (Figure 6, Table 6). The cervical and horizontal pull headgear appliances tended to induce buccal crown movement, reflecting a lateral tipping effect. Conversely, the pendulum appliance promoted palatal displacement of the root, producing a mild constrictive tendency. The AMDA and Beneslider maintained balanced displacement across the transverse dimension, while skeletal anchorage devices again demonstrated the most stable and symmetrical pattern. Such control in the transverse plane highlights their biomechanical advantages in maintaining arch coordination under the conditions simulated in the present model.

## 4. Discussion

This finite element analysis provides a comparative biomechanical evaluation of how anchorage design and force vector orientation influence the three-dimensional movement of maxillary molars. By modeling six distalization systems under identical geometric and material conditions, the study enables a controlled comparison of the biomechanical behavior of each appliance while minimizing patient-related variability. The findings show that the height and transverse position of the force vector relative to the molar’s center of resistance play a far greater role in determining movement patterns than the absolute force magnitude or appliance type. Although the general tendencies of these systems may seem predictable from basic mechanics, this study provides quantitative evidence that refines our understanding by measuring the degree of crown–root divergence, vertical control, and transverse balance produced by each design. In doing so, it bridges theoretical biomechanics with numerical data, offering clinicians a practical guide for appliance selection and vector adjustment in daily practice.

Our FEA is a static, immediate-response model; it does not capture time-dependent tissue remodeling. Accordingly, the reported displacements represent the initial response to force application and do not simulate sequential activation stages, cumulative distalization, or time-dependent anchorage loss. Material properties and boundary conditions were idealized: material properties were assumed to be homogeneous and isotropic, which may not perfectly replicate clinical reality. Nonetheless, the comparative design remains valuable for differentiating mechanics across appliances. Additionally, forces were applied using device-specific magnitudes and vectors derived from published protocols (Table 2). As a result, absolute displacement magnitudes may reflect differences in both force level and appliance design; thus, comparisons in this study emphasize 3D displacement patterns and crown–root differentials (tipping tendency) rather than displacement magnitude alone.

Recent developments in 4D FEA and clinical CBCT superimpositions provide complementary dynamic and in vivo perspectives that can refine future models [34,35]. Additionally, the dental and bony geometry was based on idealized anatomy rather than patient-specific CBCT segmentation; therefore, findings should be interpreted primarily as comparative displacement patterns under standardized conditions rather than individualized clinical predictions.

The displacement values reported here are sub-millimetric (Table 4, Table 5 and Table 6) because they reflect the immediate elastic response to a single activation in a deterministic model. Clinically, molar distalization with pendulum-type mechanics is typically in the millimeter range over months (e.g., mean M1 distalization ≈ 4.52 mm over ≈5.98 months), and systematic evidence reports mean distalization of 2–6.4 mm with distal tipping of 6.67–14.50° [32]. Accordingly, the principal clinical value of this study lies in the comparative 3D displacement patterns and crown–root differentials (tipping tendency) and the vertical/transverse components, which inform appliance selection and force vector design rather than predicting treatment duration or achieved millimeters of movement.

### 4.1. Sagittal Displacement

The present finite element analysis demonstrated distinct sagittal displacement patterns among the six appliances. The AMDA showed controlled distal movement with relatively limited tipping. The Beneslider achieved effective distalization but with greater root mesialization, echoing findings by Kinzinger et al. [12]. The pendulum demonstrated pronounced distal crown tipping, in line with Kinzinger’s early reports [12] and clinical outcomes reported by Yordanova [36]. Cervical headgear produced the largest distal tipping moments, whereas horizontal pull headgear reduced but did not eliminate tipping. These results suggest that skeletal anchorage systems may provide more controlled molar displacement patterns under the simulated conditions of this finite element model and are consistent with recent FEA comparing traction sites with clear aligners (palatal OMI > buccal OMI > Class II elastics) [37] and with IZC-based total-arch distalization mechanics [38,39].

### 4.2. Vertical Displacement

Vertical effects differed across appliances. Pendulum mechanics produced measurable molar intrusion, as reported previously [12], while cervical headgear showed extrusion with potential clockwise mandibular rotation in hyperdivergent patients [12]. AMDA and Beneslider displayed minimal vertical change, consistent with clinical acceptability [18]. Skeletal anchorage (IZ miniscrews and miniplates) maintained superior vertical control in our model, which is supported by total-arch distalization FEA demonstrating that a higher anterior force-application point reduces incisor extrusion [38] and by skeletal anchorage application-region effects on displacement vectors [39].

### 4.3. Transverse Displacement

Headgear mechanics were associated with buccal displacement, while the pendulum showed palatal tipping—patterns noted in experimental/clinical reports [12,40]. AMDA and Beneslider exhibited moderate transverse effects. Skeletal anchorage (IZ/miniplate) minimized buccolingual deviations, aligning with FEA evidence that IZC-anchored mechanics and palatal OMI traction provide better transverse control than Class II elastics [37,38,39].

### 4.4. Tipping and Root Control

Cervical headgear produced the steepest crown–root differentials, followed by pendulum, confirming limited root control [12,18]. highlights their biomechanical superiority in maintaining arch coordination. Horizontal pull headgear improved but did not normalize root control. Beneslider and AMDA showed moderate tipping control. Skeletal anchorage appliances (IZ/miniplate) provided the most favorable crown–root ratios, approaching bodily displacement [41,42]. Contemporary FEA and CBCT-based clinical assessments confirm that aligner-based distalization under routine protocols tends toward distal tipping and buccal inclination, with achieved movements smaller than predicted [34,35].

### 4.5. Clinical Implications

The observed differences have direct implications for appliance selection:

- **Skeletal anchorage** may be preferable when bodily molar movement and minimal side effects are priorities, especially in non-growing patients or cases requiring large distalization.
- **Cervical headgear** can be effective but is best suited to hypodivergent cases where extrusion is not a concern and patient compliance can be ensured [43]. Note that conventional appliances rely on patient compliance for full effectiveness; this factor can introduce variability that is beyond the scope of the static model.
- **Pendulum appliances** should be used with caution in cases where tipping or transverse constriction would be detrimental.
- **Beneslider** and **AMDA** may provide a compromise between compliance-free mechanics and acceptable control, making them versatile in moderate distalization cases. It should be noted that recent FEA studies report some differing outcomes; these discrepancies likely reflect differences in model assumptions and warrant further investigation.

## 5. Conclusions

Within the limitations of this finite element model, this study demonstrates that the three-dimensional displacement behavior of maxillary first molars is primarily governed by the height and direction of the applied force vector relative to the tooth’s center of resistance, rather than by the absolute force magnitude or appliance design alone. The findings indicate biomechanical advantages for skeletal anchorage systems and the AMDA under the conditions simulated in the present model. Appliances with higher or adjustable force vectors tended to produce more controlled distalization patterns and improved vertical control compared with conventional tooth-borne mechanics. In contrast, conventional appliances—particularly the cervical headgear and pendulum—produced more pronounced crown tipping and extrusion due to their low force vectors acting below the center of resistance. Additionally, the model evaluated only the right first molar in isolation; bilateral symmetry and full-arch effects were not analyzed and should be examined in future studies. Finally, the computational results have not been directly validated against clinical CBCT or in vitro experiments, which should be addressed in future work.

From a clinical standpoint, these findings reinforce the importance of vector control and anchorage selection in planning non-extraction Class II corrections. Skeletal anchorage and adjustable systems such as the AMDA should be prioritized when bodily molar movement and vertical control are essential, while conventional mechanics may remain suitable for compliant, low-angle patients where minor extrusion is acceptable.

Methodologically, the unified finite element framework used in this study provides a reproducible platform for comparing distalization systems under identical boundary and material conditions. Future investigations integrating dynamic (4D) modeling and patient-specific morphology could further enhance the predictive accuracy of such simulations and bridge the gap between numerical modeling and clinical performance. Contemporary evidence from FEA and CBCT superimposition studies reinforces the present conclusions on traction site selection, staging-related tipping, and real-world movement accuracy during distalization.

## Figures and Tables

**Table 1:** Material properties of the model components.

Component	Young’s Modulus (MPa)	Poisson’s Ratio
Teeth	20,300	0.30
Periodontal ligament	0.669	0.49
Trabecular bone	13,700	0.38
Cortical bone	34,000	0.26
Brackets/TADs	200,000	0.30
Orthodontic Wire	200,000	0.30
Miniscrew implants	105,000	0.33
Arch wire/Hook	2.1 × 10^5^	0.30

**Table 2:** Force magnitudes applied per appliance.

Appliance	Force Magnitude	Reference
Headgear (cervical)	2.5 N = 250 g	Geramy et al. [14]
Headgear (horizontal)	150 g	Feizbakhsh et al. [30]
Pendulum (bone-anchored)	150 g	Kang et al. [16]
Pendulum (dental-anchored)	200–250 g	Hilgers [31]
Infrazygomatic mini-implants	Open coil 200 g	Khan et al. [25]
AMDA	Open coil 150 g	Papadopoulos et al. [32]
Bollard miniplates	Open coil 200 g	Sugawara et al. [33]
Beneslider	2.4 N = 244 g	Longerich et al. [23]

**Table 3:** Characteristics of the six distalization approaches.

Appliance	Approach	Anchorage Type	Force Application	Mechanics
Headgear (Cervical/Horizontal pull)	Conventional	Tooth-borne	Buccal	Sequential
Pendulum	Conventional	Tooth-borne	Palatal	Sequential
Infrazygomatic MIs + fixed appliances	Non-compliance	Bone-borne (miniscrews)	Buccal	En-masse
Bollard Miniplates + fixed appliances	Non-compliance	Bone-borne	Buccal	Sequential
AMDA with miniscrews	Non-compliance	Bone-borne	Palatal	Sequential
Beneslider with miniscrews	Non-compliance	Bone-borne	Palatal	Sequential

**Table 4:** Sagittal displacement of the maxillary first molar along the apico-occlusal path (mm). Positive values indicate distal movement; negative values represent mesial movement. Node 1: mesio-buccal root apex, Node 49: mesio-buccal cusp tip.

Node	Cervical HG	Horizontal HG	Pendulum	IZ Mini-Implants	Mini-Plate	AMDA	Beneslider
1	−0.00541	−0.00186	−0.01119	0.00207	0.00244	−0.0003	0.00029
5	−0.00107	0.00081	−0.00962	0.00256	0.00214	−0.0002	−0.00005
10	0.00436	0.00414	−0.00766	0.00317	0.00177	−0.00007	−0.00048
15	0.0098	0.00747	−0.00568	0.00377	0.00138	0.00006	−0.0009
20	0.01524	0.0108	−0.00365	0.00437	0.001	0.0002	−0.00132
25	0.02068	0.01412	−0.00157	0.00495	0.0006	0.00034	−0.00175
30	0.02611	0.01744	0.00052	0.00553	0.0002	0.00048	−0.00217
35	0.03155	0.02076	0.00262	0.00612	−0.0002	0.00063	−0.00259
40	0.03698	0.02407	0.00471	0.0067	−0.0006	0.00077	−0.00301
45	0.04241	0.02738	0.00681	0.00727	−0.001	0.00092	−0.00343
49	0.04676	0.03003	0.00849	0.00774	−0.0013	0.00103	−0.00377

**Table 5:** Vertical displacement along the mesio-distal occlusal path (mm). Positive values: intrusion; negative values: extrusion. Node 1: distal fossae, Node 49: mesial fossae.

Node	Cervical HG	Horizontal HG	Pendulum	IZ Mini-Implants	Mini-Plate	AMDA	Beneslider
1	0.00406	0.00442	0.00126	−0.00048	−0.00351	−0.00197	−0.00210
5	0.00309	0.00382	0.00053	−0.00042	−0.00321	−0.00238	−0.00212
10	0.00189	0.00306	−0.00039	−0.00036	−0.00282	−0.00290	−0.00213
15	0.00068	0.00231	−0.00131	−0.00029	−0.00244	−0.00342	−0.00215
20	−0.00053	0.00155	−0.00224	−0.00022	−0.00206	−0.00394	−0.00217
25	−0.00174	0.00080	−0.00316	−0.00015	−0.00168	−0.00446	−0.00219
30	−0.00295	0.00004	−0.00408	−0.00008	−0.00129	−0.00498	−0.00221
35	−0.00416	−0.00071	−0.00500	−0.00001	−0.00091	−0.00550	−0.00223
40	−0.00537	−0.00147	−0.00592	0.00006	−0.00053	−0.00602	−0.00224
45	−0.00657	−0.00222	−0.00685	0.00013	−0.00014	−0.00654	−0.00226
49	−0.00754	−0.00283	−0.00758	0.00018	0.00016	−0.00695	−0.00228

**Table 6:** Transverse displacement (mm) of maxillary first molars under six distalization protocols. Negative values indicate palatal displacement; positive values represent buccal displacement. Node 1: distal fossae, Node 49: mesial fossae.

Node	Cervical HG	Horizontal HG	Pendulum	IZ Mini-Implants	Mini-Plate	AMDA	Beneslider
1	−0.04587	−0.03080	0.02409	−0.01796	−0.01366	0.02956	0.01376
5	−0.04406	−0.02942	0.02046	−0.01713	−0.01306	0.02815	0.01294
10	−0.04180	−0.02769	0.01592	−0.01608	−0.01231	0.02648	0.01198
15	−0.03955	−0.02597	0.01139	−0.01504	−0.01157	0.02483	0.01103
20	−0.03729	−0.02425	0.00685	−0.01400	−0.01082	0.02314	0.01005
25	−0.03503	−0.02252	0.00231	−0.01295	−0.01008	0.02145	0.00907
30	−0.03277	−0.02080	−0.00223	−0.01191	−0.00933	0.01976	0.00810
35	−0.03051	−0.01908	−0.00677	−0.01086	−0.00859	0.01807	0.00712
40	−0.02825	−0.01735	−0.01131	−0.00982	−0.00784	0.01638	0.00614
45	−0.02599	−0.01563	−0.01584	−0.00877	−0.00710	0.01468	0.00517
49	−0.02418	−0.01425	−0.01948	−0.00794	−0.00650	0.01333	0.00439

## Data Availability

The original contributions presented in this study are included in the article. Further inquiries can be directed to the corresponding author.
